# End to end stroke triage using cerebrovascular morphology and machine learning

**DOI:** 10.3389/fneur.2023.1217796

**Published:** 2023-10-24

**Authors:** Aditi Deshpande, Jordan Elliott, Bin Jiang, Pouya Tahsili-Fahadan, Chelsea Kidwell, Max Wintermark, Kaveh Laksari

**Affiliations:** ^1^Department of Biomedical Engineering, University of Arizona, Tucson, AZ, United States; ^2^Department of Mechanical Engineering, University of California, Riverside, Riverside, CA, United States; ^3^Department of Radiology, Stanford University, Stanford, CA, United States; ^4^Department of Medical Education, University of Virginia, Inova Campus, Falls Church, VA, United States; ^5^Department of Neurology, Johns Hopkins University School of Medicine, Baltimore, MD, United States; ^6^Department of Neurology, University of Arizona, Tucson, AZ, United States; ^7^Department of Neuroradiology, MD Anderson Center, University of Texas, Houston, TX, United States; ^8^Department of Aerospace and Mechanical Engineering, University of Arizona, Tucson, AZ, United States

**Keywords:** stroke, CNN—convolutional neural network, stroke outcome, collateral circulation, segmentation (image processing), machine learning, cerebrovascular disease

## Abstract

**Background:**

Rapid and accurate triage of acute ischemic stroke (AIS) is essential for early revascularization and improved patient outcomes. Response to acute reperfusion therapies varies significantly based on patient-specific cerebrovascular anatomy that governs cerebral blood flow. We present an end-to-end machine learning approach for automatic stroke triage.

**Methods:**

Employing a validated convolutional neural network (CNN) segmentation model for image processing, we extract each patient’s cerebrovasculature and its morphological features from baseline non-invasive angiography scans. These features are used to detect occlusion’s presence and the site automatically, and for the first time, to estimate collateral circulation without manual intervention. We then use the extracted cerebrovascular features along with commonly used clinical and imaging parameters to predict the 90 days functional outcome for each patient.

**Results:**

The CNN model achieved a segmentation accuracy of 94% based on the Dice similarity coefficient (DSC). The automatic stroke detection algorithm had a sensitivity and specificity of 92% and 94%, respectively. The models for occlusion site detection and automatic collateral grading reached 96% and 87.2% accuracy, respectively. Incorporating the automatically extracted cerebrovascular features significantly improved the 90 days outcome prediction accuracy from 0.63 to 0.83.

**Conclusion:**

The fast, automatic, and comprehensive model presented here can improve stroke diagnosis, aid collateral assessment, and enhance prognostication for treatment decisions, using cerebrovascular morphology.

## Introduction

Stroke is a major cause of death and disability in the United States and worldwide. About 800,000 people in the US suffer from a stroke each year, leading to 140,000 deaths (1). Critically reduced regional cerebral blood flow during an acute ischemic stroke (AIS) initiates brain dysfunction and, if left untreated, brain tissue death at a high rate of 1.9 million neurons every minute (2). Therefore, fast and accurate AIS diagnosis is essential for timely treatment to limit constantly growing ischemic core and irreversible brain damage (3), and improve functional outcomes (4).

In the acute hospital setting, AIS triage consists of baseline patient assessment followed by imaging studies to rule out brain hemorrhage, localize vessel occlusion sites and identify salvageable tissue-at-risk. However, the current clinical process relies on the immediate availability of vascular neurology and neuroradiology expertise, which varies significantly across institutions (4, 5). In recent years, software solutions have been developed for the automatic detection of large vessel occlusion (LVO) and estimation of the ischemic core and at-risk tissue (4, 5) using features such as the difference between left versus right hemispheric average vessel density (4, 6, 7). These approaches, however, are limited by low sensitivity (63%–85%) or low specificity (50%–70%), despite achieving high sensitivity (83%–92%) due to pre-existing brain and cerebrovasculature changes such as intracranial atherosclerosis and tandem occlusions. Therefore, developing fast, reliable, and accurate methods that can automatically extract and analyze complex cerebrovascular morphology can improve stroke diagnosis even in smaller facilities without access to local expertise (4, 8, 9).

After the stroke diagnosis is established, acute reperfusion therapies, including intravenous thrombolysis, and increasingly, endovascular thrombectomy (EVT), are used for emergent recanalization of the occluded vessels (2, 10). Accumulating evidence has established the effectiveness of EVT in improving the ischemic core to tissue-at-risk (penumbra) ratio and long-term functional outcomes (2). Hence, accurate and rapid patient selection is critical yet clinically demanding and is typically performed using a combination of clinical and imaging parameters. The most common parameters include patient baseline functional status, symptoms onset time, stroke severity, baseline non-contrast computed tomography (CT) scan or magnetic resonance (MR) imaging of the brain, non-invasive vascular imaging modalities CT and MR angiography (CTA and MRA), as well as more advanced perfusion brain imaging in selected cases (11). However, not all AIS patients are eligible for acute reperfusion therapies. In addition, response to acute reperfusion therapies is highly variable among AIS patients and relies on patient-specific cerebrovascular anatomy that governs cerebral blood flow during ischemia and reperfusion (12). Data-driven outcome prediction of acute reperfusion therapies can assist stroke systems of care in facilitating the triage and transfer of AIS patients and decision-making by physicians, patients, and their families. Therefore, approaches to automatic and individualized response prediction to treatment and outcomes are consequently gaining popularity (13, 14).

The collateral circulation consists of a network of secondary vessels developed over time to maintain cerebral perfusion and prolong brain tissue survival during ischemia (15). Accordingly, the optimal treatment window varies between individuals, and a better-developed collateral circulation provides the patient more time to receive acute reperfusion therapies (16). Therefore, a validated and rapid assessment of collateral circulation and potentially other complex cerebrovascular features can tremendously impact patient selection for treatment (17, 18). The collateral index (CI) is a metric to quantify the extent of collateral circulation development. It has been shown to significantly impact patient recovery after recanalization and long-term functional outcomes (15). However, manual CI assessment is time- and labor-intensive and not incorporated into the routine stroke triage for patient selection and outcome prediction (19).

Machine learning (ML) algorithms have been increasingly used in recent years to improve multiple aspects of stroke care, including diagnosis, treatment, and outcome prediction (20). The performance of ML algorithms depends on the input data, its structural design, and defined outcomes. Many previous attempts have been limited by the variety and reliability of their input, which typically includes parameters similar to those used for EVT patient selection, such as the National Institute of Health Stroke Scale (NIHSS) (21) and the Alberta Stroke Program Early CT Score (ASPECTS) of the baseline non-contrast head CT scan (22). However, only a very few ML models have used more advanced neuroimaging parameters (11, 23), and most have failed to incorporate patient-specific cerebrovascular features in their models, thus missing out on exploiting the rich vascular information (14, 24, 25) despite their significant impact on patient outcomes (2, 9, 26). Not surprisingly, the outcome prediction studies have reported a relatively low specificity and sensitivity with an area under the curve (AUC) for the receiver operating characteristic curve (ROC curve) under 0.76 (27, 28).

We hypothesized that incorporating patient-specific cerebrovascular morphological features would improve stroke diagnosis and long-term outcome prediction. A major challenge in the automatic estimation of the CI and other morphological features of the cerebral vasculature is the lack of a validated method for vascular segmentation and feature extraction from baseline CTA or MRA scans. In the past few years, there have been significant advances in automatic cerebrovasculature segmentation methods, which refers to partitioning an image into multiple segments to separate the regions of interest from the background, i.e., “vessels” and “non-vessels,” by assigning a label to each image pixel (29–31). We previously developed a novel, validated algorithm for accurate segmentation as well as geometric feature extraction of the cerebral vessel network (32). However, the segmentation must be extremely fast, and produce vessel network maps in real-time, for clinical applications. For this purpose, deep learning techniques are gaining popularity as they enable instantaneous 3D segmentation of volumetric imaging data. Most deep learning efforts in literature have used small datasets for neural network training and applied threshold-based vascular maps as the ground truth to generate binary vessel trees (30, 33). These limitations lead to inaccuracies in the final segmentation due to insufficient training data, the inconsistent nature of the thresholding-based approach, and low inter-observer agreement in manually segmented ground truth.

**Figure 1:**
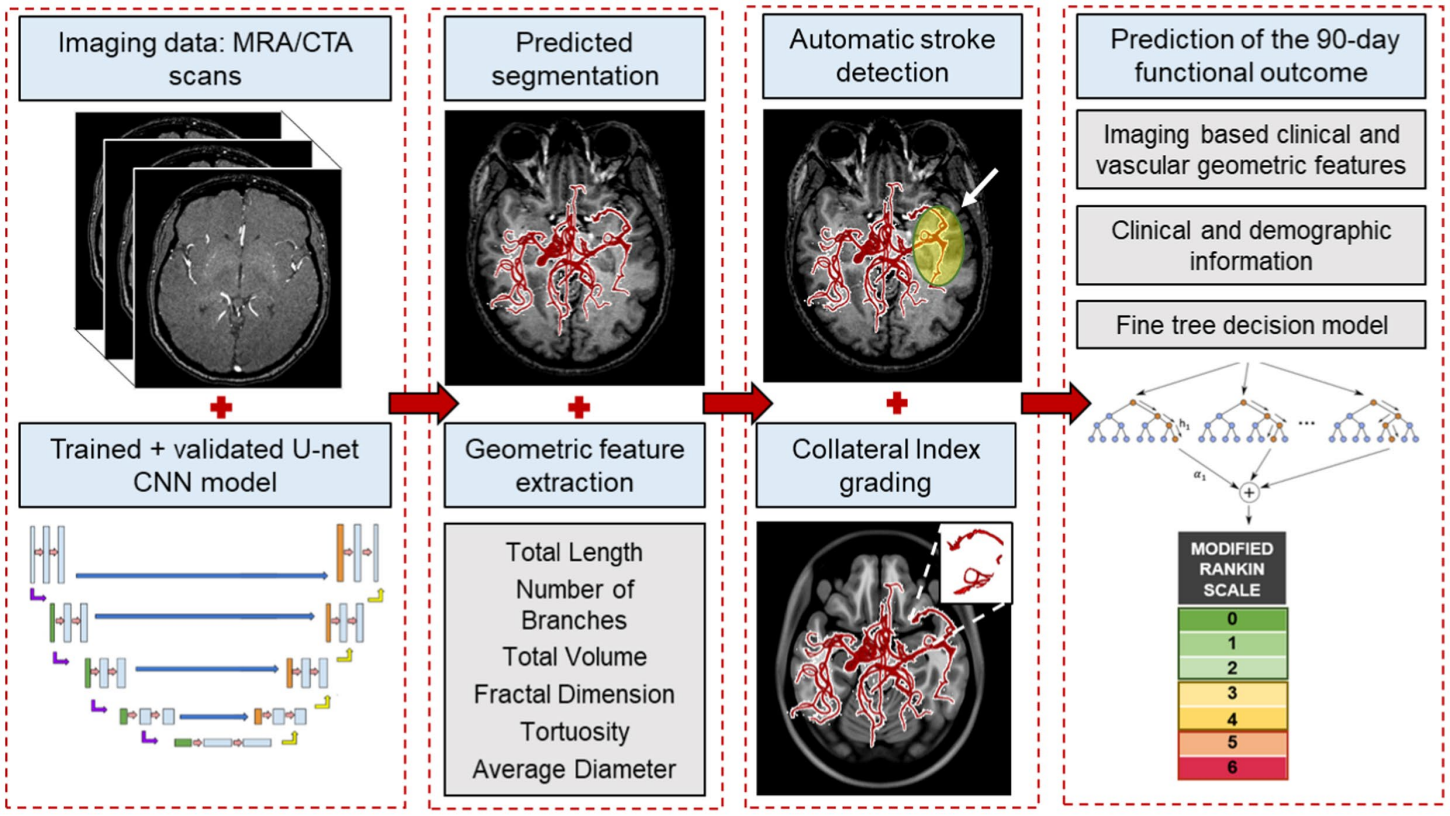
An overview of the end-to-end machine learning process. The process starts with automatically segmenting raw imaging scans using the CNN model, followed by extracting morphological features from the vessel tree. These features, along with our labeled cerebrovascular atlas of healthy adults, are used for automatic stroke detection and estimating the collateral index. Lastly, in combination with the patient’s baseline clinical and imaging data, these features are used in a fine-trees decision model to predict 90 days functional outcomes.

In this pilot study, we present a novel hybrid image processing and artificial intelligence pipeline for stroke patient triage. Figure 1 depicts an overview of our end-to-end process. Using our validated segmentation algorithm as ground truth (32), we train a convolutional neural network (CNN) for instantaneous, accurate, and automatic segmentation of the vascular network from the raw CTA or MRA scans that aids in subsequent extraction of the complex geometric features of the cerebral vessel network, namely—total length, average diameter, branching pattern, total volume, vessel tortuosity, and fractal dimension. We then used these cerebrovascular morphological features in conjunction with our previously developed statistical cerebrovascular atlas (34) to automatically detect the presence and site of the vessel occlusion, and calculate the CI for each stroke patient. The findings were validated against ground truth clinical assessment and grading of each subject’s collateral circulation. Lastly, we developed a predictive ML algorithm to incorporate the automatically extracted cerebrovascular features and CI in combination with the commonly used clinical and imaging parameters to predict the 90 days functional outcome of AIS. With this work we aim to improve various aspects of stroke patient triage by bringing imaging data to the forefront of treatment decisions.

## Materials and methods

Figure 1 illustrates an overview of the entire workflow, starting from the CNN-based segmentation of cerebrovasculature to predicting the 90 days outcome after AIS. This retrospective study uses multiple anonymized datasets, each approved by the corresponding IRB. Table 1 lists the details of all the imaging data used in this study.

**Table 1:** The imaging datasets and the corresponding number of scans used in the various aspects of the study.

Databases			Number of subjects			
Name	*N* (female)	Resolution (mm^3^)	CNN (train/test)	Stroke detection	CI validation	Outcome prediction
MIDAS (healthy)	109 (57)	0.5 × 0.5 × 0.5	99/10	50	—	—
OASIS3 (healthy)	66 (29)	0.3 × 0.3 × 0.3	41/25	—	—	—
Stroke	100 (54)	0.5 × 0.5 × 0.5	0/10	100	56	100
CTA (healthy)	10 (4)	0.43 × 0.43 × 0.62	0/10	—	—	—

The time-of-flight MR angiography (ToF-MRA) was the modality used in all databases except the healthy CTA database, where CT angiography was used. The CI estimation algorithm was validated against clinical ground truth for 56 patients and later applied to the 44 remaining patients. Additionally, 12 of the 100 stroke patients were excluded from the outcome prediction due to unavailability of their mRS outcome.

To train and test the CNN segmentation algorithm, we used time-of-flight (ToF) MRA scans of 175 healthy subjects consisting of 109 subjects from the MIDAS public database (CASILab at the University of North Carolina, Chapel Hill, NC; distributed by Kitware, Inc.) and 66 subjects from the OASIS-3 study (Knight Alzheimer Research Imaging Program at Washington University, St. Louis, MO) (35). An Allegra 3 T MR scanner (Siemens Medical Systems Inc., Germany) was used for data acquisition in MIDAS database with 0.5 mm^3^ resolution. The scans in the OASIS-3 database were acquired on three different Siemens scanner models: a 1.5 T scanner with a 16-channel head coil and two 3 T scanners with a 20-channel head coil with 0.3 mm^3^ resolution. Furthermore, Stanford University School of Medicine provided head CTA scans of 10 healthy subjects, acquired on a GE Lightspeed scanner at 100–120 kV after a bolus injection of 90–120 mL contrast media (Isovue-370 mg/mL) at the injection rate of 4–5 mL/s with 0.5 mm^3^ resolution.

We also obtained MRA scans of 100 AIS patients from the Centre Hospitalier Universitaire Vaudois in Lausanne, Switzerland, acquired on a 1.5 T Siemens scanner with 0.5 mm^3^ resolution. All patients had a confirmed diagnosis of AIS due to an occlusion in the anterior circulation in the internal carotid artery (ICA), the middle cerebral artery (MCA) at the M1, M2, or M3 segments, or a combination of these. The MRA scans were performed within 72 h from stroke onset. The database also included demographic, clinical, and additional neuroimaging data, including ASPECTS and perfusion mismatch volume and ratio, as well as 90 days functional status assessed using the modified Rankin scale (mRS), when available. We used the MRA scans and patient data to extract the patient-specific vascular features and collateral index and train our outcome prediction model. Out of the 100 AIS patients, 12 had to be excluded from the prediction model due to the unavailability of their mRS outcome.

Multiple datasets were used in the various parts of this study—175 MRA scans of healthy subjects, 10 MRA scans of stroke patients and 10 CTA scans of healthy subjects to train and test the convolutional neural network (CNN) model; 100 MRA scans of stroke patients and 50 MRA scans of healthy subjects to develop and validate the stroke detection algorithm; 56 MRA scans of stroke patients to develop and validate the CI estimation algorithm; and lastly, extracted data and from 100 MRA scans of stroke patients for the outcome prediction model development and testing.

### Segmentation and feature extraction

For CNN-based cerebrovascular segmentation, we adapted and optimized the U-Net architecture. The U-Net framework, presented by Ronneberger et al. (36), has previously been successfully used for various medical image segmentation applications. Our U-Net CNN model consisted of 18 double convolution layers, 9 in each encoding and decoding segment (Figure 1; Supplementary material). Out of the total 175 healthy MRA, 10 stroke MTA and 10 CTA scans, we used 140, 25, and 30 scans for training, validation, and blind testing, respectively with a stratified sampling of the CNN segmentation model. This was done to ensure a division into 80% for training and 20% for test data from each database and overall. We used our previously validated methodology for segmentation and vascular feature extraction to obtain the “ground truth” of the segmented vascular maps and their respective features (32). The validated segmentation algorithm involves a multi-step process, resulting in a 3D binary volume of the vessel network, which is then skeletonized to obtain the centerlines of the vessels and the diameter at every point on the centerline, with being able to detect vessels with diameters as small as the imaging resolution. It tracks connected tubular structures in 3D volumes. It thereby performs better than other available segmentation algorithms under technical issues that can cause intensity inhomogeneities in imaging data, such as motion artifacts and inflow effects. After segmentation, we performed a skeletonization of the vessel network. A “branching node” of the vascular tree, defined as a point connected to 3 other points (i.e., a bifurcation), was used to calculate the global geometric features of the vessel tree as follows:

1. Total length: the total length of the vessel network is calculated by summing the skeletal segments’ length between 2 nodes.
2. Total number of branches: a “branch” was defined as a sequence of points along the vessel beginning at a bifurcation point and ending either at the next bifurcation or the last point on the vessel (i.e., a terminating branch).
3. Average and maximum branch length: all network branches’ mean length (geodesic distance) and the longest branch found.
4. Average diameter: the mean diameter at all points on the centerline.
5. Total volume: volume of the vessel network is calculated by considering the vessels as cylinders with varying diameters along the total length.
6. Fractal dimension: the fractality of the vessels was determined using the box-counting method. This feature is a measure of morphological complexity in cerebral vasculature.
7. Vessel tortuosity: vessel tortuosity was defined using the sum of angles measurement between sets of 3 points on the centerline divided by the total length.

For CNN training, the raw scans were the model input and mapped to their corresponding segmented vascular networks, using the above method as output. The model architecture includes skipping connections built in between the encoding/contraction and the mirrored decoding/expansion path for each scale level, with deconvolutional layers replacing the 2-stride convolutions. We use a small kernel size of 3 × 3, and our model consists of 18 total layers—9 double convolution layers each in the encoding and decoding segments. We implement the rectified linear unit (ReLU) activation function throughout the layers and a sigmoid function at the last layer for the final prediction of grayscale pixel intensities between 0 and 1, corresponding to vascular pixels as opposed to the background. Adam optimizer was used for the gradient descent, and the model was trained for 50 epochs until the loss was minimized. Early-stopping and drop-outs were used as additional regularizers to prevent overfitting and improve generalization (37). We defined an application-specific loss function known as “Dice loss,” which, when minimized, maximizes the overlap of segmentation prediction and ground truth, measured using the Dice similarity coefficient “SoftDice” (38).

For CNN validation and testing, we compared the model predictions against the segmented vascular maps of the test data obtained by the vessel segmentation algorithm (ground truth) using an image matching/registration metric, namely the Dice similarity coefficient (DSC). The DSC quantifies the overlap between the ground truth and the prediction for each slice of the 3D volume as a measure of accuracy and is obtained by averaging the DSC per 2D cross-sectional slice across each 3D volume and then averaging across all the test scans. Also, as a further quantitative assessment of model performance, we compared the following extracted features obtained by the CNN model against the ground truth: total length, number of branches, total volume, and average diameter. We calculated the error margins for these features as an additional indicator of segmentation accuracy. Lastly, the CNN model was tested on the CTA of 10 healthy subjects and MRA of 10 AIS patients to accurately evaluate the model’s ability to segment vasculature in different modalities and health status.

### Stroke detection and occlusion localization

**Figure 2:**
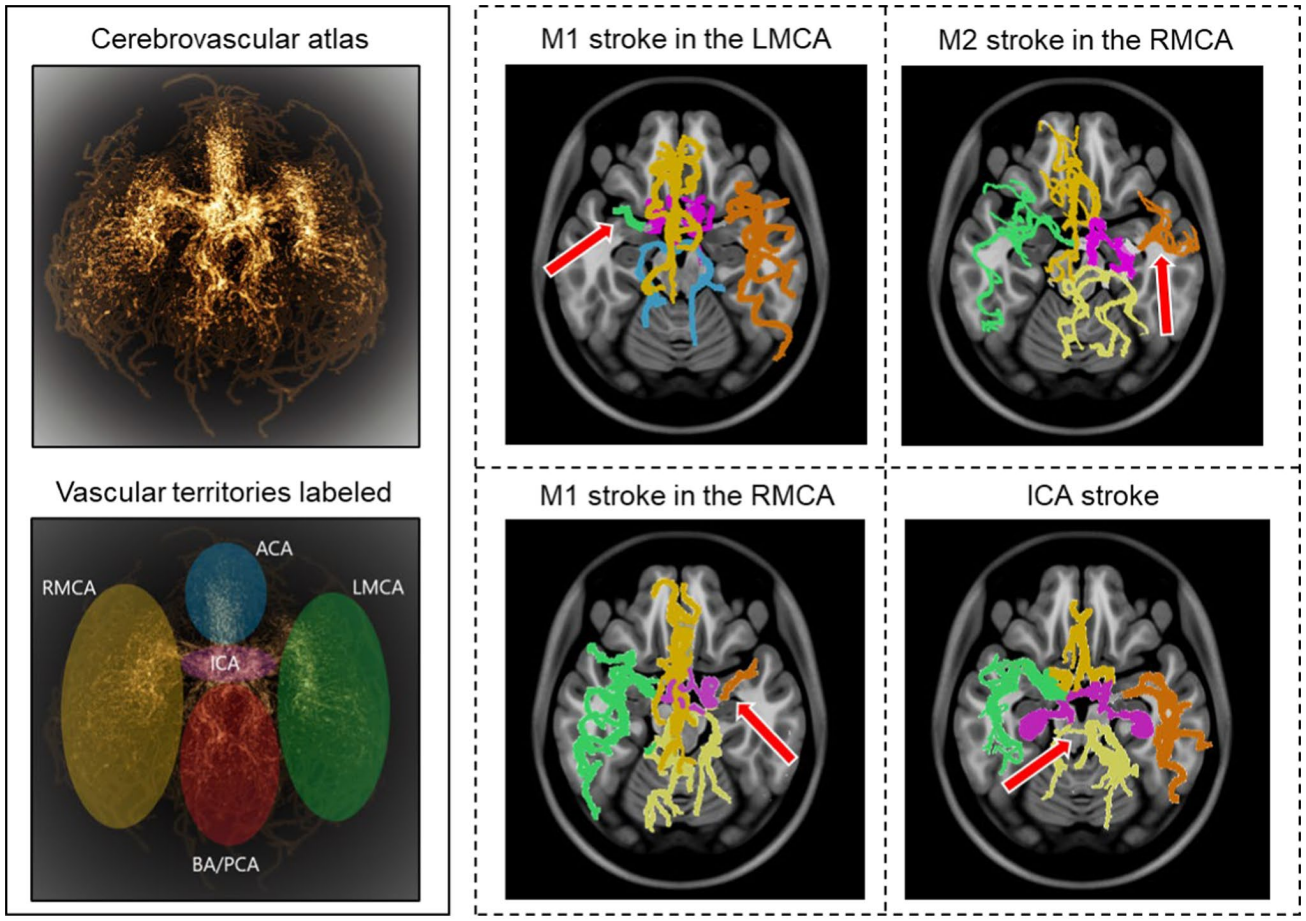
Stroke detection and occlusion localization. The brighter regions of the three-dimensional probabilistic atlas (top−left) show maximum intensity projection and correspond to a higher probability of vessel occurrence. The brain vascular was divided into five major territories, illustrated in different colors (bottom−left). The cerebrovasculature map of four stroke patients is shown on the right. The red arrow indicates the location of the occluded vessel.

Of 100 AIS patients, the occlusion sites were the internal carotid artery (ICA) in 18, tandem ICA-MCA in 17, and M1 and M2 segments of the MCA in 46 and 19, respectively. We utilized our previously developed probabilistic cerebrovascular atlas of spatially co-registered cerebral vessel maps of 175 healthy adults (34). The atlas was labeled to denote the five major vascular territories: (1) the ICA, (2) and (3) the left and right MCA, (4) anterior cerebral artery (ACA), and (5) the posterior cerebral artery (PCA) and basilar artery (BA), as illustrated in Figure 2.

We implemented a two-step approach for automatic stroke detection based on previous preliminary studies that showed a significant difference in cerebrovascular features between stroke patients and healthy subjects (32, 34). In the first step, the presence of occlusion was determined by comparing the vessel density of the patient-specific vascular network with the probabilistic cerebrovascular atlas using the total vessel length, volume, and the number of branches. A significantly lower vessel density, defined as more than three standard deviations below the average healthy subjects, indicated an occlusion. In the second step, each of the five vascular territories of AIS patients was compared with the corresponding territory in the probabilistic atlas. The vascular territory with the largest average vaso-deviation score from the healthy average was identified as the occlusion location (Figure 2). We also compared the vessel density between the left and right cerebral hemispheres for each patient to ensure that the algorithm can also detect occlusion in case the comparison against the atlas was not sensitive enough. The analysis was performed for all 100 AIS patients, and 50 randomly selected healthy subjects to assess the algorithm’s ability to detect the occlusion’s presence and location. We evaluated the algorithm’s performance by calculating both steps’ sensitivity, specificity, and positive and negative predictive values (PPV and NPV).

### Automatic collateral index estimation

The collateral index (CI) quantifies the development of a patient’s collateral vessel network. In clinical settings, using different scales, CI is usually graded as either good, intermediate, or poor. The clinical grading which was utilized for developing and validating our method is as follows (39):

0 = collateral supply absent

1 = collateral supply filling >0% but ≤50%

2 = collateral supply filling >50% but <100%

3 = 100% collateral supply

To provide a fast and automatic calculation of CI, we used our previously developed probabilistic cerebrovascular atlas in conjunction with the extracted patient-specific vascular geometric features (34). Of the 100 AIS patients included in the study, we had access to the CI for 56, graded in the clinic by a neuroradiologist, since this metric is not routinely estimated in all stroke patients due to the time constraints and manual nature of the task. These clinically-evaluated CIs were used as the “ground truth” to develop and validate our algorithm. We then use the validated method, described below, to obtain the CI for the remaining 44 AIS patients automatically.

The relative vessel density in the collateral region was calculated for each patient compared to patients with the fully developed collateral network (CI = 3). Based on this relative index obtained using linear regression, we grade the collaterals per patient on a scale of 0 (absent collateral supply) to 3 (100% developed collateral supply). We used this automatically estimated CI for the 100 patients as an outcome predictor in our ML model, given the well-established impact of collateral circulation on the patient’s response to ischemia and eventual treatment (15, 16, 40).

### Functional outcome prediction

The functional status of the AIS patients was evaluated at the 90 days mark using the modified Rankin scale (mRS) (Table 1; Supplementary material) (41). The input data for the prognostication model comprised of patients’ clinical and imaging variables, including demographic information, pertinent past medical history, stroke symptoms and severity (the baseline NIH stroke scale or NIHSS), and data from initial brain imaging studies, including the Alberta Stroke Program Early CT Score (ASPECTS) and perfusion metrics (the volume of the ischemic core and tissue-at-risk and their mismatch ratio). The perfusion mismatch ratio refers to the ratio of the ischemic core to the critically hypo-perfused tissue-at-risk (penumbra) that is commonly measured by the cerebral blood volume versus the mean transit time or time-to-maximum of the blood flow. A larger mismatch volume or ratio indicates a larger salvageable tissue that may be amenable to acute treatments (42–44).

Cerebrovascular geometric features have been shown to correlate with aging and pathologic states (32, 34, 45–47). The vessel structure and geometry, including lumen diameter and branching patterns, are known to impact the patient’s response to ischemia and reperfusion (12, 15, 48). To utilize the predictive ability of the rich imaging-based vascular information, we also incorporated novel patient-specific cerebrovascular geometric features extracted by the segmentation algorithm described above (32) as well as automatically estimated CI into our ML model as additional predictors of the 90 days mRS. Table 2 lists all the features used as predictors to train the model.

**Table 2:** CNN model performance on test data per geometric feature between the predictions vs. ground truth.

Validation metric	Value (mean ± standard deviation)
Avg. DSC	0.94 ± 0.05
Avg. error in total length (%)	3.40 ± 0.31
Avg. error in the number of branches (%)	1.90 ± 0.44
Avg. error in total volume (%)	3.18 ± 0.26
Avg. error in average diameter (%)	2.11 ± 0.10

To assess the performance of the ML model predictions, the in-clinic assessment of the mRS at 90 days post-stroke was considered the ground truth. In this study, we trichotomized the 90 days mRS scores into good (mRS 0–1), moderate (mRS 2–3), and poor (mRS 4–6) outcome groups. Reducing the 7-class mRS to the three output classes will facilitate better prediction and provides a more granular classification than the dichotomized predictions in literature, since there are oftentimes higher number of misclassifications between good vs. moderate and moderate vs. poor outcomes (14, 20, 25). We used supervised ML to train the predictor model using the Classification Learner app in the MATLAB^®^ package (Mathworks, MA). The dataset was divided into 80% training and 20% test datasets for validation. We also included stratified sampling and five-fold cross-validation to improve learning and prevent overfitting. The features were then ranked using the chi-square test based on the univariate associations between each categorical or continuous predictor variable and the 90 days mRS outcome. The ranked features were used as predictors, and the three final outcome groups were the ground truth for the output classes. The final outcome prediction model was chosen based on the training and validation performance metrics; the optimal model for our dataset was determined to be the fine trees decision model. The model performance was assessed by calculating the accuracy using the true positive and false negative rates and the positive and negative predictive values. The findings were visualized by the AUC of the ROC curve. Since we are performing a multi-class prediction, the AUC was computed for the ROC curves using the one-against-rest method for multi-class models (49).

## Results

### CNN segmentation model

For rapid, real-time segmentation, we developed a convolutional neural network (CNN) using a U-net architecture (36) to extract the cerebral vessel network from raw imaging scans, leveraging our previously validated algorithm as segmentation “ground truth.” We used 140 MRA scans of healthy subjects for training and tested the model on four separate datasets consisting of 55 subjects: 35 MRA scans of healthy subjects (10 from the MIDAS dataset with 0.5 mm^3^ resolution and 25 from the OASIS3 dataset with 0.3 mm^3^ resolution), 10 MRA scans of AIS patients with 0.5 mm^3^ resolution, and 10 CTA scans of healthy subjects with 0.5 mm^3^ resolution (Table 1).

**Figure 3:**
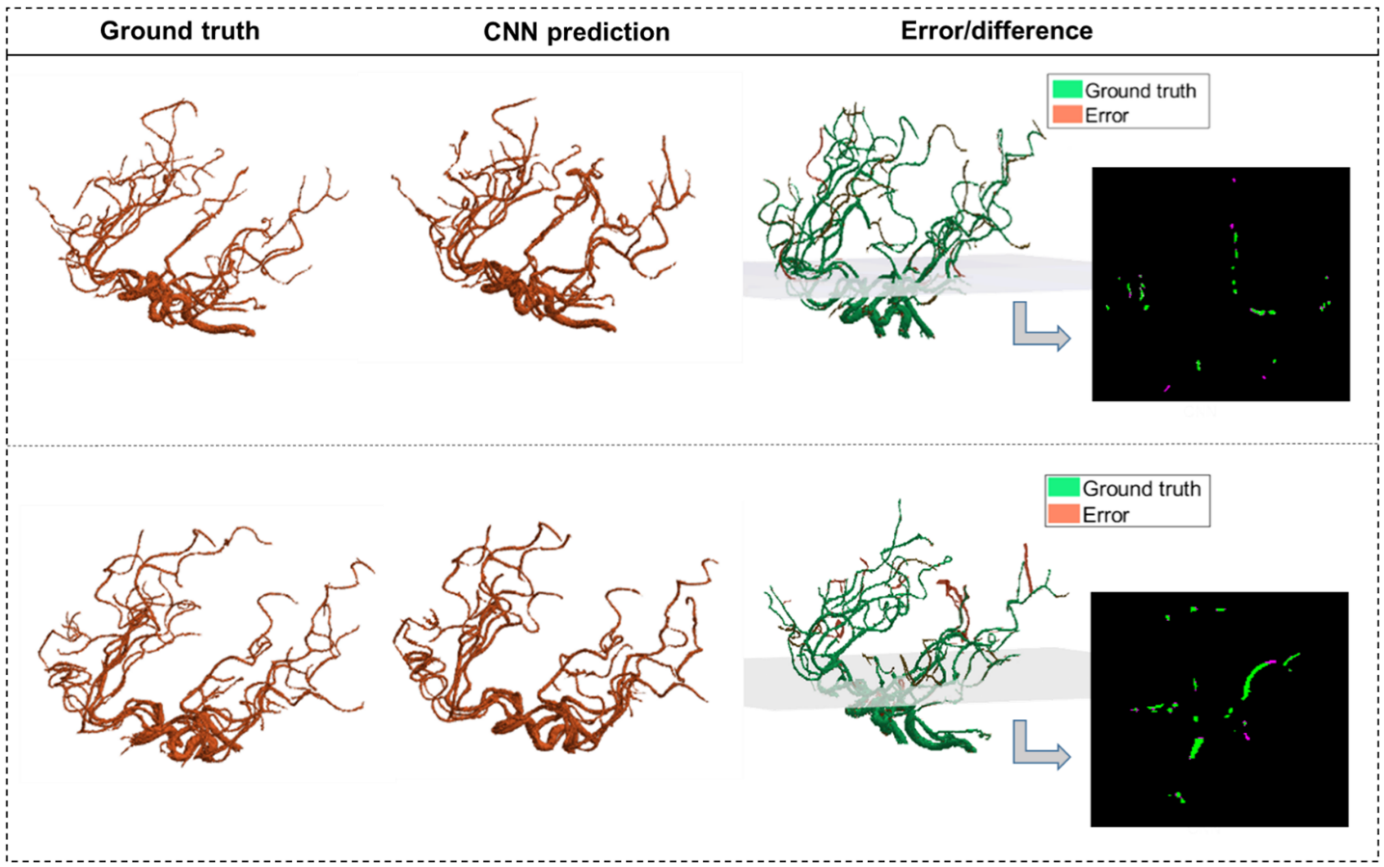
The CNN predictions of the segmented vascular maps. CNN segmentations of two MRA scans from test data are shown alongside the corresponding ground truth segmentations obtained using our validated algorithm. The third column shows the error volume overlaid on the ground truth volume, with one 2D panel showing the overlap between corresponding slices of the ground truth and the error.

The average Dice similarity coefficient (DSC) between the ground truth segmentation and CNN predictions across the multi-resolution test data was 0.94. This was obtained by averaging axial slice DSC values of each subject and then averaging across all subjects. To further validate that the model captures the vascular branches and preserves the volume, the segmented vascular features were compared against the ground truth. The average error margins were under 4% for all geometric features: total length (3.4%), number of branches (1.90%), total volume (3.18%), and average diameter (2.11%). Figure 3 shows two predicted segmentation maps and their corresponding ground truth. The quantitative measures of model performance on the test dataset are shown in Table 2.

### Stroke detection and occlusion localization

Our model identified 92 strokes among 100 AIS patients and 50 healthy subjects with a sensitivity of 92% and specificity of 94%. The algorithm only missed the presence of an LVO in 8 cases due to the overall vessel length and volume not being significantly different than the atlas due to the presence of significant vessel density in the proximal part of the middle cerebral artery (MCA) in case of M2 strokes, in the distal segment of the MCA (Figure 2). Additionally, the algorithm detected three false positives among the 50 healthy subjects due to the vascular networks deviating significantly from the atlas.

The model had an accuracy of 95.6% in identifying the region containing the occlusion, with only four mis-localizations out of the 92 stroke cases identified. These errors are due to outliers in vascular patterns deviating from the average atlas. Overall, the model achieved a 95.56% positive predictive value (PPV) and a 95.56 negative predictive value (NPV).

### Collateral index calculation

**Figure 4:**
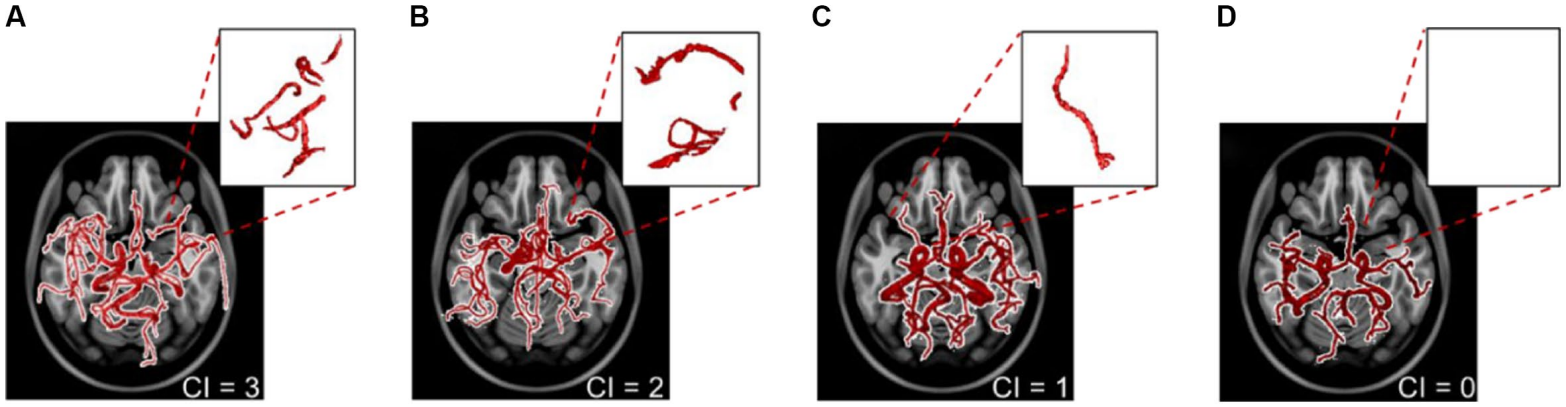
The extracted vascular networks and estimated collateral index for the four varying levels of development of the collateral network in patients with a middle cerebral artery stroke. The collateral index (CI) is shown at the bottom right of panels **(A–D)**. The top right sub-panels show the vessels in the corresponding collateral region of the ipsilateral hemisphere.

We used cerebrovascular morphologic features and the probabilistic cerebrovascular atlas for the automated scoring of CI from poor or absent collateral circulation to a fully formed collateral network. Our method was validated against clinically graded CI for 56 out of the 100 stroke patients and was then applied to the remaining patient scans. The algorithm correctly estimated the CI in 49 out of the 56 strokes MRA, yielding a sensitivity of 87.2%. Figure 4 shows the vascular tree, extracted vessel network from the ipsilateral collateral region, and the estimated CI for 4 cases, each corresponding to a CI score from 0 to 3.

### Functional outcome prediction

**Figure 5:**
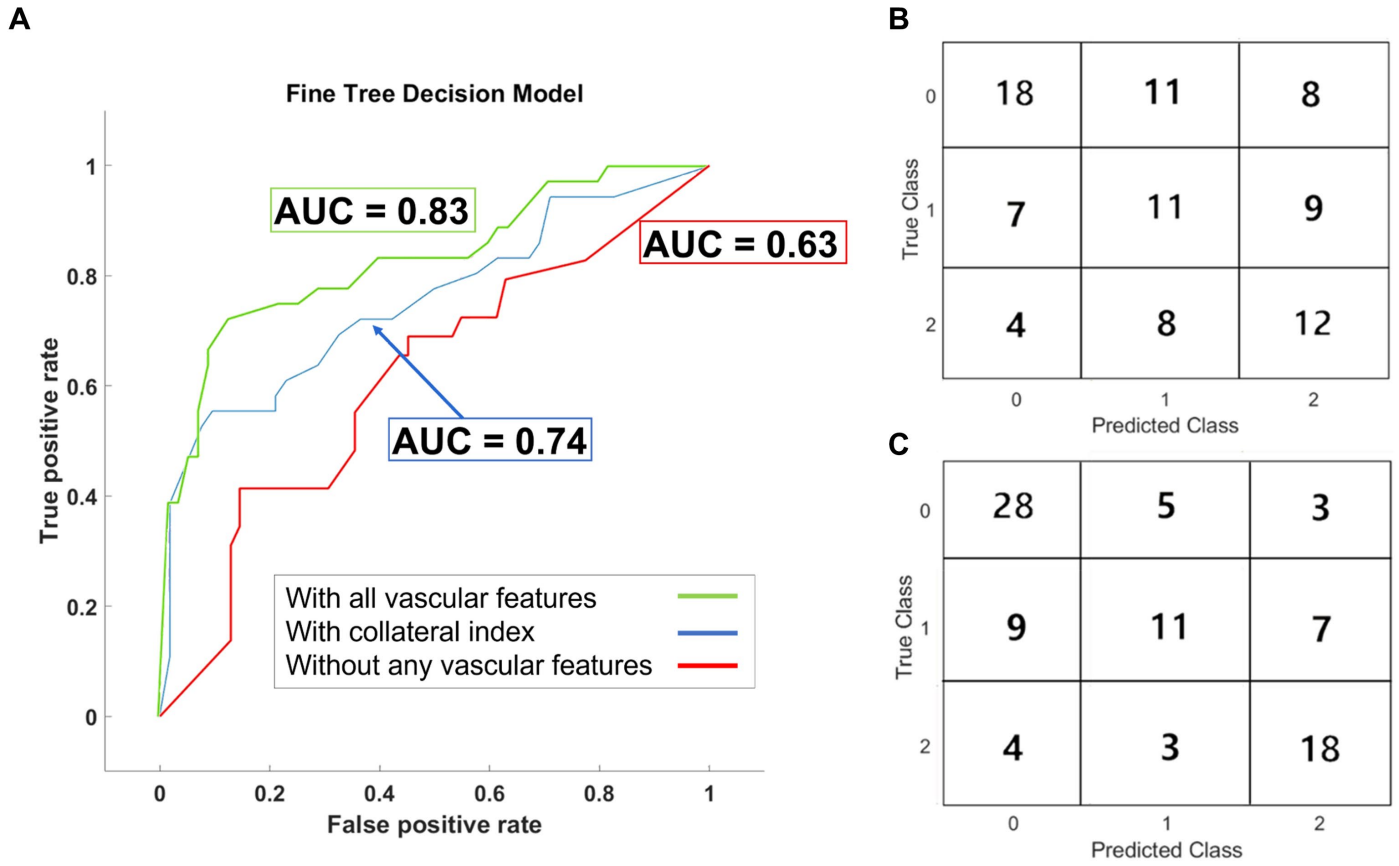
Results of the outcome prediction models. **(A)** ROC curves for the three fine decision tree models are plotted. All three models included the baseline clinical and imaging data. AUC-ROC for the model without any vascular features was 0.63. AUC-ROC increased to 0.74 in the model trained with the collateral index but without other geometric features. Incorporating all vascular features improved the model’s performance with AUC-ROC =0.83. The confusion matrix for the prediction model before **(B)** and after **(C)** incorporating the vascular geometric features and auto-estimated collateral index are shown.

Fine decision trees were adopted for multi-class prediction of the 90 days modified Rankin scale (mRS) (41). The basic predictor model using conventional predictors of functional outcomes (Table 3), reached an area under the curve (AUC) of the receiver operating characteristic (ROC) curve of 0.63 ± 0.01 (Figure 5A), similar to currently available models. We then investigated the effect of incorporating cerebrovascular morphologic features to the model, to improve prognostication after AIS. By including the automatically estimated CI in the prediction model, the and the AUC of the ROC curve increased to 0.74 ± 0.02. The additional incorporation of vascular geometric features further increased the prediction accuracy with an AUC of the ROC curve of 0.83 ± 0.02 (Figure 5A). ROC curves in Figure 5 are overlaid on the same graph to highlight the AUC values, with the true class being the “Good” outcome (i.e., mRS 0–2). The confusion matrices (Figures 5B,C) show the prediction accuracy per outcome group for the predictive models with and without the morphologic features.

**Table 3:** The clinical and imaging variables used to train the prediction models.

Clinical and demographic information	Imaging-based clinical features	Vascular geometric features
Age	ASPECTS score	Total length
Sex	Side of the stroke	Number of branches
Baseline NIHSS	Occlusion location	Total volume
History of stroke	Perfusion mismatch ratio and volume	Average diameter
Smoker (y/n)	Collateral index (CI)—ground truth	Tortuosity
Diabetes mellitus (y/n)		Fractal dimension
Hypertension (y/n)		Auto-estimated CI

NIHSS, National Institute of Health Stroke Scale; ASPECTS, Alberta Stroke Program Early CT Score. The cells in blue represent the conventionally used features, yellow refers to the automatically estimated CI using our method and green cells represent the novel vascular predictors.

## Discussion

Machine learning (ML) strategies have been used in various applications in stroke medicine (9, 14). In this work, we presented an end-to-end automatic ML approach for stroke triage, consisting of a CNN-based cerebrovascular segmentation and morphologic extraction, an automated algorithm for stroke detection and collateral circulation assessment, and finally, a 90 days functional outcome predictor.

### Cerebrovascular segmentation using CNN

Accurate and efficient segmentation of brain vascular imaging by extraction and visualization of the 3D cerebrovascular network is critical for clinical practice. We had previously developed and validated a method to detect brain vessels as small as the image resolution (voxel size) with superior performance compared to other freely available segmentation software (32). However, depending on the image resolution and computing resources, our previous model took up to 20–30 min to segment one vascular imaging study. Here, we used the segmented vascular maps extracted by our algorithm as the ground truth to train a U-Net architecture-based CNN model for accurate and instantaneous segmentation of 3D vessel networks from raw imaging data.

U-Net has widely been accepted as the gold standard for image segmentation applications, more so in the medical image processing field. Its widespread use is justified by its optimized and flexible modular design and the ability to perform well for all kinds of imaging modalities (50). Medical images like ours can involve complex vascular structures of interest at varying scales, making segmentation challenging. The U-Net architecture has been specially designed to segment precisely these kinds of multi-scale complex structures and has been shown to out-perform other kinds of neural networks for similar medical image segmentation tasks (50, 51). Additionally, the customizability offered by U-Nets, such as the skip-connection enhancements we use in our work, further elevates the applicability and performance of this versatile and easy to implement model for our application. U-Net models require relatively significantly smaller number of training data points as compared to other networks that need thousands of training data samples to learn (51).

The existing CNN-based segmentation methods require manual annotation with increased “noise” in the final segmented map pertaining to the erroneous prediction of non-vessel voxels from the skull or bright spots on the scans (30, 33). The U-Net CNN model employed in this study demonstrated accurate segmentation without requiring manual annotations. It detects the finer vessels present in the ground truth segmentation with a small error margin and, in some cases, detects smaller tapering vessel voxels that were not present in the ground truth segmentation of the blind test data due to poor contrast in the raw imaging scan at those voxels. The segmented vascular edges are smooth, and the CNN predictions do not miss vessel pixels around the boundary surface of the vessel cross-sections, as seen in 2D slices, resulting in the diameter and other geometric features to be computed accurately.

### Stroke detection and occlusion localization

Vascular morphology differs significantly between healthy and stroke subjects (32, 34). Implementing complex cerebrovascular features and quantitative measurement of vaso-deviation from the average healthy forms the basis of our stroke detection algorithm. Using a labeled atlas of healthy vessel networks and their inherent geometrical properties, we identified the anatomical region of occlusion in the most commonly occurring ischemic strokes. The sensitivity of our algorithm is comparable to (and in some cases higher than) previously published methods, and the specificity has improved as well (4, 5). The high sensitivity and specificity of the stroke detection algorithm demonstrate the applicability of vascular geometry in automated stroke diagnosis and occlusion localization rather than a simplistic hemispheric comparison that may lead to false detections due to inconsistent vascular symmetry between the two hemispheres. Early diagnosis of AIS and identifying the occluded vessel can facilitate acute treatments and enhance the efficiency of stroke care systems. Such a tool could be invaluable in radiological screenings in case of an emergency or lack of on-call neuroradiologists in smaller medical centers.

### CI estimation and functional outcome prediction

A large number of studies support the significant benefits of endovascular thrombectomy in treating acute ischemic stroke (1, 2, 10, 11, 52). The eligibility for EVT is expected to expand, along with technological advancements, with a shift from rigid time-based treatment protocols to imaging-based strategies that incorporate patient-specific factors into therapeutic decision-making, such as collateral circulation (2). Our previous study of healthy human cerebrovasculature and the development of a probabilistic vascular atlas (34) showed that the Circle of Willis is not fully formed in most adult humans, and a significant cerebrovascular variability exists within the population. These variations affect cerebral hemodynamics and even rates of neuronal degradation (53, 54) during ischemia and, thereby, response to treatment. Hence, a more developed collateral circulation provides more time (even several days) for therapeutic interventions and impacts clinical outcomes (15, 16). Therefore, a better understanding of each patient’s cerebrovasculature and collateral circulation are pivotal to expanding eligibility for acute treatments (17). The method presented in this study can accurately, rapidly, and automatically calculate the collateral index from cerebrovascular morphologic features. This development can drastically impact patient triage and reduce the time for diagnosis and treatment (55).

Stroke is a complex and multifaceted disease, and its underlying pathophysiology is yet to be fully discovered. Prognostication of AIS remains challenging (24) despite its tremendous impact on decision-making for patients, their families, clinicians, and society (27, 56). An algorithm that can achieve early, reliable, and accurate prognostication is lacking. Many previous attempts at using ML for outcome prediction after AIS have yielded low-performance algorithms with low sensitivity and AUC for ROC curves under 0.76 (24, 25, 28, 56). This study presents a novel ML approach incorporating quantitative cerebrovascular information to predict the functional outcome. Adding the automatically graded CI as a predictor significantly improved the 90-outcome prediction, shown as a 17% higher AUC of the ROC curve. Including other vascular geometric features further enhanced the predictive utility of the algorithm, as shown in Figure 5. Our current model is designed and tested to detect acute ischemic stroke in large vessel occlusions (LVOs) and can be possibly expanded to distal medium vessel occlusions. But if the cerebral ischemia is being caused by a (symptomatic) stenosed vessel, in most cases the carotid, our model has not yet been optimized or trained for carotid disease. It should also be noted that our current model is focused on prognosis and is agnostic to the etiology of stroke. In future iterations of the model, we aim to include this information in order to better understand its effects on patient outcomes.

### Limitations

Post segmentation, the stroke detection algorithm calls for a comparison with the cerebrovascular atlas, which, in turn, requires the patient-specific vascular network to be spatially co-registered to the Montreal Neurologic Institute (MNI) space, utilized to normalize all patient scans spatially. However, the standardized MNI atlas space is intended for MR scans, and CT scan images cannot be co-registered to the same space. Thus, for a wider appositeness of this method, CT scan data needs to be registered to this common space. Additionally, due to the inherently distinct nature of subject-specific vascular anatomy, patient vascular network alignments sometimes differ significantly from the probabilistic atlas in the cartesian space. This can cause errors in stroke detection as well as collateral estimation algorithms.

As a pilot study, we retrospectively analyzed a smaller stroke patient database (*n* = 100) to establish our methods and the utility of cerebrovascular morphology in stroke diagnosis and prognostication. Outcome prediction typically requires a large and representative patient database to assess predictive features accurately. Our training dataset was also limited to patients with large vessel occlusion in the anterior circulation. Furthermore, patients with missing outcomes were excluded from the final model (*n* = 12). A future large and prospectively collected dataset of patients with various stroke syndromes, including those with strokes in the posterior circulation and medium or small vessel occlusion, is necessary to solidify the effectiveness of vascular geometry as a predictive tool in AIS patients.

## Conclusion

The rapid and automatic process presented here aims to improve the accuracy and efficiency of detecting and localizing intracranial vessel occlusion and the fidelity of predicting the functional outcomes of stroke. We presented a novel end-to-end quantitative machine-learning strategy to extract patient-specific cerebrovascular morphology accurately, rapidly, and automatically from segmented vessel trees, automatically calculate the collateral circulation index, and predict 90 days functional outcomes. The model can improve patient outcomes by aiding diagnosis and facilitating enhanced patient selection for stroke treatment. Our method for automatic CI grading can help address the incongruity between the significant impact of collateral circulation assessment in AIS patients and the lack of time and resources to perform this task in the acute hospital setting. Through this approach, we highlight the need for patient selection and treatment decisions to be based on quantitative, imaging-based information along with clinical patient evaluation Future large prospective studies are warranted to further establish the role and applicability of the model.

## Data availability statement

The original contributions presented in the study are included in the article/Supplementary material, further inquiries can be directed to the corresponding author.

## Ethics statement

This study uses retrospective data from previously IRB approved public datasets, collected at multiple institutions. The manuscript details this information as well. The studies were conducted in accordance with the local legislation and institutional requirements. Written informed consent for participation was not required from the participants or the participants’ legal guardians/next of kin because this study uses retrospective data from previously IRB approved public datasets, collected at multiple institutions. The manuscript details this information as well.

## Author contributions

AD and KL: conceptualization and methodology. AD and JE: software and investigation. AD: visualization and writing—original draft. BJ and MW: resources. KL: funding acquisition, project administration, and supervision. AD, KL, JE, BJ, MW, PT-F, and CK: writing—review and editing. All authors contributed to the article and approved the submitted version.
