# Supplementary material for: End to end stroke triage using cerebrovascular morphology and machine learning
Source: Front Neurol. 2023 Oct 24;14:1217796. doi: 10.3389/fneur.2023.1217796 (PMC10628321; doi:10.3389/fneur.2023.1217796)
Supplement: Supplementary file 1 [file Table_1.DOCX]

**Supplementary Table 1.** **The Modified Rankin Scale** used in clinics to assess functional outcomes in stroke patients at 90 days post recanalization. This is the final output from the prediction model to quantify patient status.

| **MODIFIED RANKIN SCALE**​ | |
| --- | --- |
| **0**​ | No symptoms​ |
| **1**​ | No significant disability despite some symptoms, able to perform all activities​ |
| **2**​ | Slight disability, unable to perform all activities but can function without assistance​ |
| **3**​ | Moderate disability requires some help, can walk without assistance​ |
| **4**​ | Moderately severe disability, unable to walk without assistance, unable to attend to bodily needs without assistance​ |
| **5**​ | Severe disability, bedridden, incontinent, requires constant nursing care and attention​ |
| **6**​ | Death​ |

**
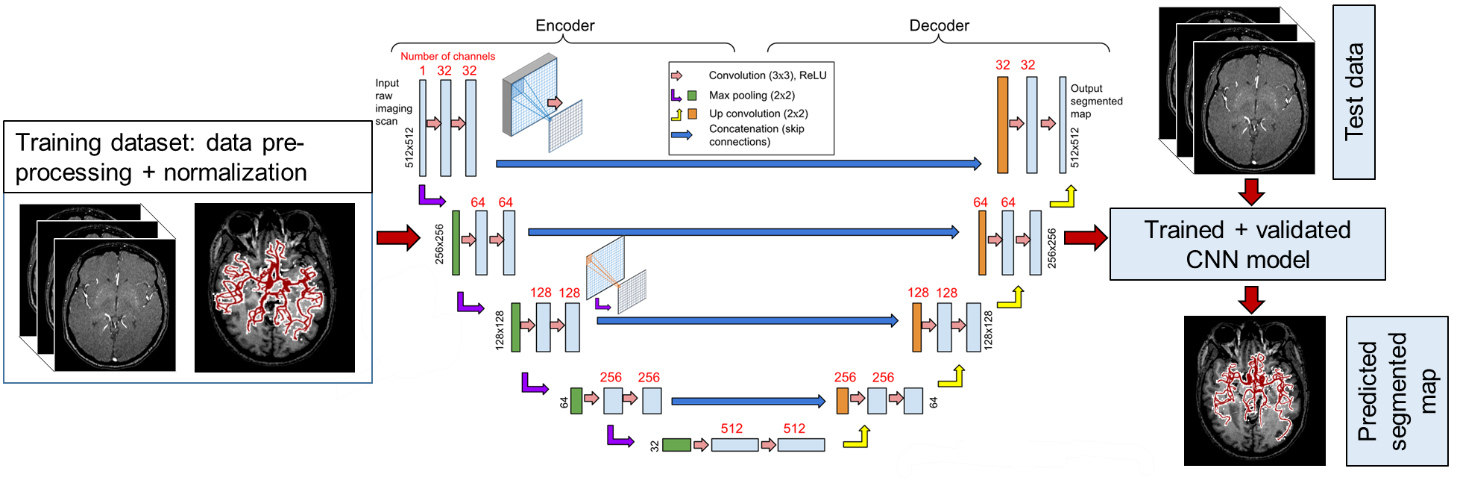
**

**Supplementary Fig. 1.** **CNN-based segmentation model.** The model is designed using the U Net architecture, consisting of 18 total double convolution layers, 9 in each encoding and decoding segment, to achieve instantaneous segmentation.
